# Feasibility of Increased Navy Bean Powder Consumption for Primary and Secondary Colorectal Cancer Prevention

**DOI:** 10.2174/1573401310666140306005934

**Published:** 2014-05

**Authors:** Erica C Borresen, Kerry A Gundlach, Melissa Wdowik, Sangeeta Rao, Regina J Brown, Elizabeth P Ryan

**Affiliations:** 1 Department of Environmental and Radiological Health Sciences; 2 Department of Food Science and Human Nutrition, Colorado State University, 1680 Campus Delivery, Fort Collins, CO 80523, USA; 3 Kendall Anderson Nutrition Center, Room 114 Gifford Building, Fort Collins, CO 80523; 4 Animal Population Health Institute, Department of Clinical Sciences, Fort Collins, CO 80523, USA; 5 University of Colorado Health, Cancer Center of the Rockies, Fort Collins, CO 80522, USA

**Keywords:** Colorectal cancer, dietary fiber, dietary practices, functional foods, legumes, navy beans, nutrition, *Phaseolus vulgaris* L.

## Abstract

**Introduction:**

Emerging evidence supports that increased consumption of dry beans (*Phaseolus vulgaris* L.) reduces both the incidence and recurrence of adenomatous polyps or precancerous growths. Navy beans have been studied for dietary colorectal cancer (CRC) chemoprevention in animal models. Our main objectives were to assess the feasibility of increased navy bean consumption in adults with and without history of CRC and to achieve intake amounts associated with chemoprevention.

**Methods:**

Seven meals and six snacks were developed for both the absence and inclusion of cooked navy bean powder (35grams/day). Sixteen healthy adults (7 non-cancer and 9 CRC survivors) completed the placebo-controlled, randomized, single-blinded dietary intervention trial. Participants consumed one study-provided meal and snack daily for 28 days, which accounted for approximately one-third of their total recommended caloric intake (meals = 202-483 kcal and snacks = 194-401 kcal). Participants also recorded three-day dietary food logs each week.****

**Results:**

The addition of 35g of cooked navy bean powder (NBP) into foods provided 5-8% daily caloric intake. The compliance to the meal and snack intervention ranged from 89-100%. Non-cancer participants in the NBP group had a significant decrease in total caloric intake after week 4 (p≤0.0001). CRC survivors in the NBP group significantly increased total fiber intake by week 4 (p≤0.0001).

**Conclusions:**

NBP are feasible to include in meals for increased total fiber intake and for consuming the amount that is associated with CRC chemoprevention outcomes. These findings warrant further evaluation of NBP consumption in clinical nutrition trials for CRC control and prevention.

## INTRODUCTION

Colorectal cancer (CRC) is the third most commonly diagnosed cancer and the second leading cause of cancer-related deaths in the United States [1]. Dietary recommendations remain a crucial component of effective CRC primary and secondary prevention [2]. The World Cancer Research Fund and the American Institute for Cancer Research (WCRF/AICR) support increased fiber intake for CRC prevention [3]. Fiber-rich foods include fruits, vegetables, legumes, and whole grains. A large body of cancer prevention research has focused on fruit and vegetable consumption, yet emerging evidence shows that legumes, specifically common dry beans (*Phaseolus vulgaris* L.), are bioactive staple foods for CRC chemoprevention [4-11]. Navy beans, when consumed above 5% of total dietary intake, have demonstrated CRC chemoprotective activity in animal studies and were associated with reduced recurrence of adenomatous polyps (a precancerous adenoma that poses a high risk for developing colorectal carcinomas if left untreated) in humans [5-7].

Navy beans are a popular variety of dry bean that provide a quality source of protein, are low in fat, and contain high concentrations of fiber (1/2 cup = almost 10 grams of total fiber) [8]. Navy beans also contain B vitamins and minerals, such as iron, calcium, copper, zinc, phosphorous, potassium, and magnesium. Bioactive navy bean compounds include, but may not be limited to, saponins, monosaccharides, disaccharides, oligosaccharides, and ferulic acid [5]. While animal studies have shown dietary navy bean mediated chemoprevention *via* reduced adenomatous polyps and changes in inflammatory cytokine levels with diets consisting of about 75% navy beans [5, 7, 11], this large amount may not be a practical recommendation for most people. Therefore, determining what constitutes an effective quantity of navy beans for daily human consumption (*e.g.* g/day) and that can elicit health beneficial effects merits investigation.

Epidemiological studies have assessed dry bean intake alongside consumption of other types of legumes in the Leguminosae family (*e.g.* dry peas, lentils, soybeans, and peanuts). Since all types of legumes are commonly grouped together during nutrient intake analysis [12-14], it can be difficult to infer the specific contribution of dry beans for chemoprevention. However, a dietary analysis of participants in the Polyp Prevention Trial did reveal a significant relationship between elevated dry bean consumption and protection against colorectal adenoma recurrence [6]. These reported levels of dry bean intake ranged between 31-233 g/day as collected *via* food frequency questionnaires, and far exceed U.S. population consumption of less than 17g/day [15].

Currently, American adults do not meet the dietary guidelines for dry bean consumption and less than 8% of the population consumes dry beans on a given day [16, 17]. These low levels of reported dietary intakes may signify the challenges, dislikes, or inconveniences for people to add dry beans into their regular daily diets. Inclusion of cooked dry bean powders into meals is considered a practical approach to increase dry bean intake because they offer the same nutrients found in cooked whole beans and are a versatile addition to diverse food types (*e.g.* crackers, cookies, soups, breads) [18]. Many studies have assessed the safety and nutritional contents following dry bean powder inclusion in ready-made foods (*e.g.* tortillas and crackers) [19-23]. Siddiq and colleagues analyzed physical and functional characteristics of various dry bean flours for widespread use in food products [23]. The major goal of this study was to determine the feasibility of incorporating cooked navy bean powders (NBP) into meals and snacks that can be used for human clinical nutrition studies aimed to assess cancer prevention and control outcomes.

A preliminary data analysis from an ongoing, placebo-controlled, randomized, single-blinded dietary intervention trial titled, Beans Enriching Nutritional Eating For Intestinal health Trial, or BENEFIT, is presented herein. BENEFIT was implemented as part of a community-academic partnership between University of Colorado Health (Northern Region) and Colorado State University for advancing dietary chemoprevention research. We hypothesized that daily consumption of cooked NBP (35g powder, equivalent to ~1/2 cup cooked whole beans) increases total fiber intake and is palatable to achieve 5-10% of total dietary caloric intake in adults. The rationale for this amount was based on the effective navy bean consumption dose determined from animal studies of carcinogenesis [5, 24].

## MATERIALS AND METHODS

### Study Design and Participant Recruitment

A four-week, placebo-controlled, randomized, single-blinded dietary intervention trial was established at Colorado State University (CSU) as part of a community-based collaboration with the University of Colorado Health (formally the Poudre Valley Health System) in Fort Collins, CO (NCT01929122). Both healthy adults with no history of cancer and CRC survivors were recruited. To be eligible for participation, CRC survivors had to be at least 4 months post chemotherapy/radiation treatment and no prior history of cancer at any other site. Inclusion criteria for all participants included no history of food allergies or major dietary restrictions, not currently pregnant or lactating, non-smokers, no antibiotic use within the last month, and no history of gallstones.

**Fig. (1):**
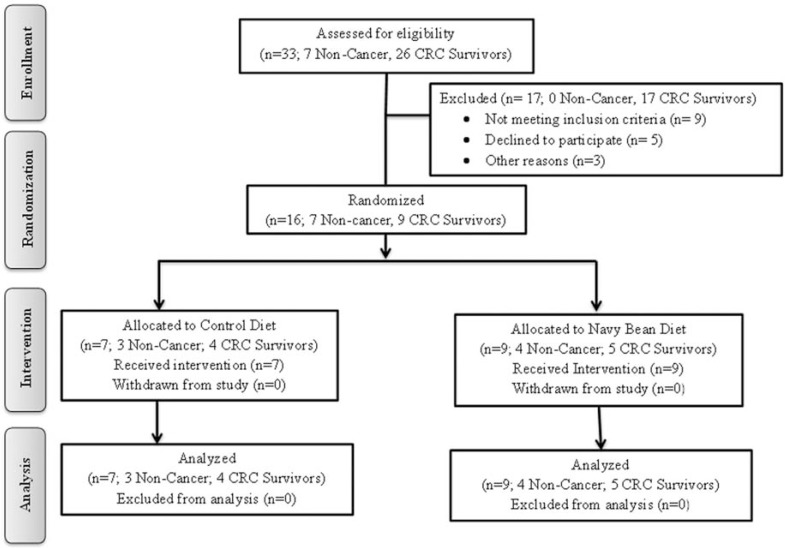
BENEFIT study participant flow.

Study participants who met the eligibility criteria were randomized based on BMI and sex. CRC survivors were recruited through the Poudre Valley Cancer Network and the University of Colorado Health. Healthy adults with no history of cancer were recruited throughout Northern Colorado through convenience sampling. Figure** 1** shows the flow of participants through the study. The CSU Research Integrity and Compliance Review Office and the University of Colorado Health Institutional Review Board approved this study protocol and informed consent form. Written informed consent was obtained from all participants prior to enrollment.

There were a total of three study visits: baseline, 2-week, and 4-week. At each study visit, participants provided stool, urine, saliva, and blood samples. The study participants were given study ID-labeled containers for self-collection of stool, urine, and saliva. These samples were collected within 24 hours of their scheduled appointment. Stool samples were analyzed for modulation of the microbiome and metabolome. Urine and saliva small molecules were analyzed using metabolomics. An experienced phlebotomist collected blood samples at the CSU Hartshorn Health Center by venipuncture. Serum lipid panel results were provided to all study participants based on the evidence for dry beans to reduce cholesterol and regulate blood lipids [25-28]. Changes in blood lipid parameters were not evaluated as primary outcomes of the study and baseline blood lipid levels were not included in randomization.

A total of sixteen participants were randomized in this phase: 7 non-cancer adults and 9 CRC survivors. Table **1** shows the baseline characteristics of the study population. All randomized participants successfully completed the pilot study and no adverse events were reported.

### Recipe Development

This dietary intervention trial consisted of two study arms: placebo-control and cooked NBP (35g/day). A registered dietitian and a certified chef developed seven meals and six snacks covering a wide range of taste preferences. Table **2** lists the names of the study meals and snacks that all participants received each week. One snack was provided twice in a 7-day period. ADM Edible Bean Specialties, Inc. provided the cooked NBP as a gift (Archer Daniels Midland Company, Decatur, Illinois). These cooked NBP (Vege Full™) originated from ground cooked navy beans and the process included washing, soaking, and cooking whole beans, prior to grinding and dehydrating to create their powder form [18]. 

Recipes for the dietary intervention meals and snacks were analyzed using Nutritionist Pro^TM^ diet analysis software (Axxya Systems, Stafford, Texas). Each intervention meal and snack contained half (17.5g) of the participant’s required daily amount of cooked NBP. Study participants achieved the total daily intake of 35g cooked NBP (equivalent to ½ cup of cooked dry beans) by consuming one study meal and one snack each day.

The placebo-control meals contained similar ingredients and appearance as the intervention meals without the addition of cooked NBP. Table **3** illustrates the nutrient profile for one meal and one snack from the control and NBP diets. The marked difference in fiber intake was intentional to examine the intake feasibility *via* cooked NBP. Similar palatability and appearance of recipes containing cooked NBP or placebo were confirmed with community taste-testing trials and in accordance with IRB protocol approvals (data not shown). 

### Dietary Intervention and Data Collection

Participants received a two-week supply of the study meals and snacks at their baseline and 2-week visits. To keep blinded to the intervention, the study meals and snacks were labeled Group A or Group B. Only the study coordinator was aware of the study arm for each participant. Participants were instructed to consume one study-provided meal and snack each day and were allowed to eat freely the rest of the day, including allowance of cooked dry beans. The dietary intervention accounted for approximately one-third of total daily caloric intake, such that each study meal ranged between 202-483 kcal and each study snack ranged between 194-401 kcal. These meals and snacks were incorporated into a 7-day meal plan that was provided to assist participants in pairing study meals and snacks, as well as to help meet total daily caloric and macronutrient needs. This meal plan was developed by a registered dietitian in accordance with recommendations set by the Institute of Medicine’s Dietary Reference Intake levels for Acceptable Macronutrient Distribution Ranges of carbohydrate (45-65%), protein (10-35%), and fat (20-35%) [29]. All participants received the meal plan to serve as a guide only and were not required to follow it verbatim.

Study compliance to the daily intervention meal and snack was determined by participants’ daily record of meal and snack consumption in increments of 25%, 50%, 75%, or 100%. Participants also completed a three-day dietary food log each week *via* recording total food intake from two weekdays (Monday-Thursday) and one weekend day (Friday-Sunday). Data analysis was completed on a total of four, 3-day food logs collected from each week on study. Food logs were analyzed using Nutritionist Pro^TM^ and each participant’s weekly dietary analysis included average daily caloric intake, macronutrient, amino acid, vitamin, and mineral profiles. 

### Calculating Percent of Navy Bean Intake 

Animal studies report navy bean intakes as a percentage of total intake, and we hypothesized that 35g cooked NBP/day would achieve the efficacious 5-10% navy bean intake in humans. The following equation was used to determine the percent of navy bean intake for each participant (consuming 35g of cooked NBP contributes 110 calories [18]): 

% navy bean consumed = [(110 kcal cooked NBP+ kcal from navy bean in regular diet)/ total average daily kcal] x 100

### Statistical Analysis 

An interim analysis was completed on the dietary intake data for sixteen participants who successfully completed the four-week trial using Statistical Analysis System (SAS) V9.3 (SAS Institute Incorporated, Cary, NC). The primary endpoint of the diet collection was change in nutrient profiles, especially fiber intake levels at the end of the study (week 4). Due to the small sample size, a non-parametric approach with Wilcoxon two sample t-test was used to evaluate significance between the baseline characteristics of the two populations (Non-Cancer = no history of cancer; CRC survivor = Colorectal Cancer Survivor). A multivariable linear regression analysis was performed to determine the effect of diet and time point on the specific calories and macronutrients. Since the sample size was small, the outcome variable was converted into ranks to perform linear regression analysis. Interaction effects of diet and time point were also evaluated. To account for clustering among the same individual measured over time, a repeated measurement approach was taken up. A priori p-value was set at 0.05 for determining statistical significance.

## RESULTS 

Participant baseline characteristics are shown in Table **1**. All sixteen participants were analyzed in this interim analysis phase that occurred between August 2010 and June 2012. Three non-cancer and four CRC survivors were allocated to the control diet. Four non-cancer and five CRC survivors were allocated to the NBP diet. CRC survivors were significantly older than the non-cancer participants as CRC is commonly diagnosed later in life (p=0.01). No other significant differences in baseline characteristics were found between the groups. 

### Feasibility of Increasing Navy Bean Powder Intake

All study participants were 89-100% compliant with regards to daily consumption of the study-provided meal and snack for 28 days. Fourteen out of the 16 participants (88%) completed three-day dietary food logs each week. At each visit, the study coordinator asked each participant to report any major issues with the diet intervention. In the event that participants mentioned gastrointestinal discomforts, they were instructed to eat their study meals in smaller portions throughout the day. The few participants (n=3) that required this adjustment were alleviated of their intestinal discomforts after 1 week on study and had no further complaints. There were no differences in the number of gastrointestinal discomforts reported between control and NBP groups. 

### Total Percent Dietary Intake of Navy Bean 

The dietary intake of 35g of cooked NBP each day resulted in 5.4-6.3% navy bean intake for the non-cancer cohort, and 5.9-8.1% navy bean intake for the CRC survivor cohort. These ranges were average percent intakes across the 4 weeks of participation. The highest weekly navy bean percent intake recorded from the non-cancer cohort was 7.4% and the CRC survivor cohort was 9.2%. The lowest weekly percent intake recorded from the non-cancer cohort was 4.6% and the CRC survivor cohort was 5.2%. Even though all participants were free to consume dry beans of any variety (*e.g.* black, red/white kidney beans, pinto), dietary food logs revealed that none of the participants were consuming the current dry bean recommendations of a half cup per day. Besides the study intervention of 35g of cooked NBP each day, the next highest consumed bean was the red kidney bean. The small amount of reported intake averaged 53 g/week or about ¼ cup of beans each week.

### Navy Bean Powder Effects on Total Caloric Intake

Dietary food log analysis revealed a significant decrease in total caloric intake at week 4 in the NBP non-cancer adult cohort (p≤0.0001) and CRC survivors in the control group (p=0.0007). Tables **4** and **5** show caloric intake changes across the groups. The median caloric intake for non-cancer adults eating cooked NBP was 1791 kcal at week 2 and decreased to 1688 kcal at week 4. The control group had a significantly higher caloric intake at week 4 (2099 kcal) compared to the cooked NBP group at this time point (p=0.002). The median caloric intake for CRC survivors on the control diet was 1961 kcal at week 2 and decreased to 1717 kcal at week 4. CRC survivors consuming cooked NBP did not see a significant change in caloric intake from week 2 to week 4 (p=0.39), where their median intake changed from 1685 kcal at week 2 to 1770 at week 4.

### Navy Bean Powder Contribution to Carbohydrate, Fat, and Protein Intake

We next analyzed changes in the macronutrients during the diet intervention. Tables **4** and **5** present the macronutrient data for the non-cancer adults and CRC survivors, respectively. The NBP non-cancer group had a significant decrease in carbohydrate intake at week 4 (p≤0.0001), where the median decreased from 245g at week 2 to 232g at week 4. Carbohydrate intake was also significantly lower at this time point compared to the control group’s median intake of 277g (p≤0.0001). No other significant macronutrient changes were seen in the NBP non-cancer group. Despite a non-significant change in caloric intake for the non-cancer control group, this group had a significant decrease in protein (p≤0.0001) and a significant increase in fat (p≤0.0001) at week 4 compared to week 2. The median protein intake decreased from 80g at week 2 to 78g at week 4, while the median fat intake was 72g at week 2 and increased to 81g at week 4. The CRC survivor participants in the control group had significantly decreased protein (p≤0.0001), carbohydrate (p=0.0016), and fat (p=0.005) intakes. The median protein intake decreased from 75g at week 2 to 59g at week 4. Median carbohydrate intake went from 232g to 197g, and median fat intake decreased from 78g to 67g at week 4. The CRC survivor group consuming NBP significantly increased carbohydrate intake with a median at week 2 of 220g to 231g at week 4 (p=0.02), the carbohydrate intake at week 4 was also significantly higher compared to the control group (p=0.04).

### Navy Bean Powder Contribution to Total Fiber Intake

**Fig. (2):**
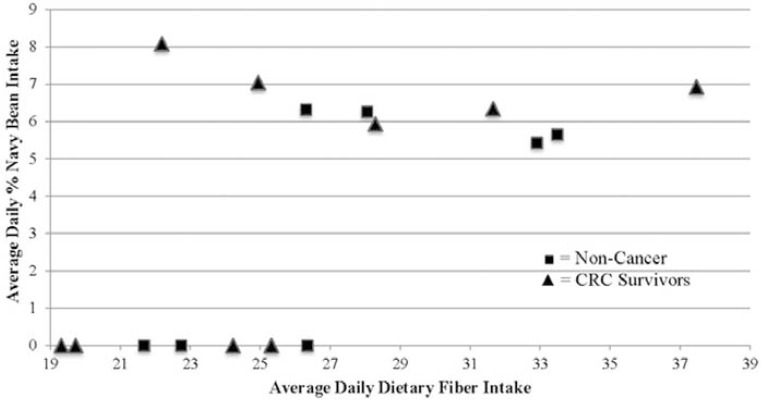
Relationship between ‰ navy bean intake and average dietary fiber intake for all participants after 4 weeks. The percent navy bean intake was calculated using the equation: *‰ navy bean consumed = [(110 kcal cooked NBP + kcal from navy bean in regular diet)/ total average daily kcal] x 100*.

An addition of 35g of cooked NBP provided 9 extra grams of fiber per day in the navy bean cohorts [18]. The only group that had a significant increase in fiber intake from week 2 to week 4 was CRC survivors consuming the cooked NBP (p≤0.0001). This group had a median intake of 27g at week 2 and increased to 29g at week 4. This fiber intake at week 4 was also significantly higher than the CRC survivor control group of 19g (p≤0.0001). Median fiber intake decreased from 23g (week 2) to 19g (week 4) for the non-cancer control group and increased from 25g (week 2) to 27g (week 4) for the non-cancer NBP group. Neither of these changes were significant (p=0.35 and p=0.54, respectively). Figure** 2 **shows the average daily percent intake of navy bean and the average daily dietary fiber intake (g/day) for all study participants to illustrate the relationship between navy bean intake and total fiber intake. Individuals in the NBP cohorts consumed higher amounts of fiber compared to the controls throughout the 4 weeks. 

## DISCUSSION

Dietary dry bean intake provides quality fiber, protein, and phytochemicals that have convincing evidence for CRC prevention in animal studies [5, 7, 11], yet Americans continue to fail to meet dietary recommendation goals for beans and total daily fiber intake [30]. This study examined inclusion of cooked NBP into seven meals and six snacks as a promising solution to increase bean and fiber consumption in adults. Our findings suggest that these powders were a viable approach and that bean powders should be next evaluated in diverse global populations with varied dietary preferences. This study demonstrated feasibility for increasing cooked NBP intake in a blinded manner and without any reported gastrointestinal discomforts in adults without a history of cancer and CRC survivors. Our findings of decreased total caloric intakes and increased fiber intakes in our NBP group compared to control group provide rationale for evaluating these effects in a longer-term cohort investigation of cooked NBP intake for primary and secondary CRC prevention. The high compliance (89-100%) to the dietary intervention further supports that 35g of cooked NBP/day was a reasonable amount for people to consume immediately, and particularly for people that were not regular consumers of dry beans. 

Calculating the total percent intake of navy beans consumed was novel and a precise means by which we can extrapolate from doses used in animal studies. Currently, there is no known recommended daily intake level for beans with regards to achieving dietary CRC chemoprevention in humans. The 35g/day of cooked NBP when incorporated into the regular diet was sufficient to achieve 5-10% total intake levels in humans (Fig. **2**), and was comparable to efficacy shown in animal models of carcinogenesis [24]. Elevated dry bean intake is certainly safe as traditional, staple dry bean consumption in Latin America and Africa can reach as high as 40+ kg annual consumption per person (~110 g/day) [31].

The ability to measure changes in flatulence and abdominal pains in a placebo controlled manner was possible due to the effective blinding of all study participants to their assigned group. This blinding component of the study design was important for the evaluation of gastrointestinal discomforts (*e.g.* excessive bloating or flatulence) because these symptoms could lead to early withdrawal from study or low compliance. Our findings confirm that of another study where concerns over gastrointestinal discomforts may be overstated, especially when individuals gradually increase dry bean consumption [32]. Winham and Hutchins found that more than half of their study participants did not report gastrointestinal distress after 1 week of consuming ½ cup of beans per day and over 70% had no symptoms after the second week on study [32]. These findings indicate that gastrointestinal symptoms should not be considered a major concern for most people when increasing bean consumption. 

The addition of study meals and snacks to healthy adults significantly decreased caloric intake after 4 weeks, and implies a satiety component to our NBP intervention. Weight control is another benefit attributed to legume consumption [33] and although we saw decreased total intakes in our cohort, there were no significant changes in weight reported after two or four weeks in any of our study participants. Future studies that extend the intervention beyond 3-6 months warrant evaluation of dry bean intake and potential for associated weight loss. Additionally, increased fiber consumption has been found to promote satiety [33]. Current dietary recommendations for fiber intake are 25-38 grams per day [29]. We significantly increased fiber intake to meet these recommendations in the CRC survivors consuming cooked NBP after 4 weeks (Table **5**). Notably, sample size was a major limitation to identify significant differences between groups during interim analyses. However, our findings for NBP effects on total caloric intake and dietary fiber intake suggest the need to evaluate participants’ satiety and eating behaviors using validated questionnaires during the dietary intervention. 

High fiber diets including dry beans have also been linked to longevity. The NIH-AARP diet and health study cohort was analyzed for sources of fiber intake associated with clinical outcomes. Park *et al*. revealed that higher consumption of dietary fiber, particularly from whole grains and legumes, had a significantly lower risk of mortality over a 9-year period [12]. Surprisingly, fiber intake from fruits and vegetables did not demonstrate a significant association. These findings provide additional support for the increased education and awareness needed regarding dry bean associated health properties, particularly in clinical nutrition and dietetics program curriculums. Additionally, the relationship between longevity and increased dry bean consumption may not only be associated with the fiber contents as other nutritional components and phytochemicals may work synergistically for health promotion and disease prevention [34-36].

## CONCLUSION

Navy beans have tremendous opportunity to improve public health nutrition across all demographics because it is an inexpensive way to increase fiber intake, promote satiety, and receive the bioactive components that may reduce colorectal cancer risk. Our intervention includes cooked NBP in prepared foods to achieve 5-10% of total dietary intake and this represents a promising strategy for CRC control and prevention in diverse populations with varied taste preferences and dietary patterns. We put forth that additional human intervention trials with cooked NBP are needed to better understand how much to consume and how this food works for CRC control and prevention globally. 

## Figures and Tables

**Table 1.** Baseline characteristics of study population.

Characteristic	Non-Cancer Adults (n=7)	CRC Survivors (n=9)
Age (years)	39.6 ± 15.1	60.9 ± 11.0*
Sex Males (%) Females (%)	3 (43%) 4 (57%)	2 (22%) 7 (78%)
BMI (kg/m2)	26.0 ± 5.3	25.9 ± 5.0
Total Cholesterol (mg/dl)	186.0 ± 40.0	192.8 ± 38.5
LDL(mg/dl)	112.7 ± 32.8	115.9 ± 32.6
HDL(mg/dl)	52.4 ± 13.2	54.4 ± 6.3
Triglycerides(mg/dl)	106.3 ± 56.6	113.7 ± 65.1
Fruit intake (X servings/wk)a 0≤X≤2 X>2	3 4	4 5
Vegetable intake (X servings/wk)a 0≤X≤2 X>2	5 2	9 0
Grain intake (X servings/wk)a 0≤X≤4 X>4	5 2	6 3

Values are presented as mean ± SD.

* Statistically significant (p ≤0.05).

a From first 3-day dietary food log.

**Table 2.** Dietary intervention meals and snacks^a^.

Meals	Snacks
Baked Pasta Marinara Butternut Squash Soup Mexican Chicken Bake Pizza Margherita Homemade Chili Tomato Basil Soup Tuna Cheddar Casserole	Banana Nut Muffins Blackberry Cobbler Caraway Crackers Cranberry Apple Granola Energy Date Bites Strawberry Pineapple Smoothie

a Study participants consumed 1 meal and 1 snack each day.

**Table 3.** Nutrient analysis of dietary intervention for one meal and one snack in each study arm

Meal Example: Mexican Chicken Bakea	Control	Navy Bean Powder
Serving size (g)	340	348
Calories (kcal)	352	368
Protein (g)	26	26
Carbohydrates (g)	30	34
Fat (g)	15	16
Saturated Fat (g)	7	8
Fiber (g)	3	8
Soluble Fiber (g)	0.1	2
Iron (mg)	3	3
Vitamin A (mcg RE)	167	173
Vitamin C (mg)	28	28
Folate (µg)	65	73
Zinc (mg)	2	3
Calcium (mg)	250	311
Potassium (mg)	616	1094
Sodium (mg)	714	721
Snack Example: Banana Nut Muffina	Control	Navy Bean Powder
Serving size (g)	115	119
Calories (kcal)	251	260
Protein (g)	7	10
Carbohydrates (g)	42	44
Fat (g)	7	8
Saturated Fat (g)	2	2
Fiber (g)	3	7
Soluble Fiber (g)	0.3	2
Iron (mg)	2	2
Vitamin A (mcg RE)	48	48
Vitamin C (mg)	4	4
Folate (µg)	45	53
Zinc (mg)	1	2
Calcium (mg)	55	103
Potassium (mg)	269	736
Sodium (mg)	124	129

a Dietary intervention meals and snacks were analyzed using Nutritionist Pro^TM^ diet analysis software (Axxya Systems, Stafford, Texas).

**Table 4.** Total calories and selected macro- and micronutrient intake at 2-week and 4 week time points for non-cancer cohort^*^.

Non-Cancer Study Population	Control Diet		Navy Bean Powder Diet	
	Week 2	Week 4	Week 2	Week 4
Calories (kcal)	2015 ± 325 (2186)	2048 ± 266 (2099)a	1967 ± 378 (1791)	1680 ± 195 (1688)a,b
Protein (g)	82 ± 14 (80)	78 ± 18 (78)b	87 ± 18 (93)	63 ± 8 (59)
Carbohydrates (g)	265 ± 54 (291)	268 ± 53 (277)a	256 ± 46 (245)	214 ± 44 (232)a,b
Fat (g)	67 ± 14 (72)	75 ± 12 (81)b	67 ± 20 (61)	61 ± 12 (54)
Fiber (g)	24 ± 3 (23)	24 ± 8 (19)	29 ± 8 (25)	26 ± 2 (27)

* Values are presented as mean ± SD (median). Medians are included since ranks were compared in the analysis.

a Significance (p≤0.05) between Control and Navy Bean Powder Groups at time point.

b Significance (p≤0.05) at Week 4 compared to Week 2 for specific diet.

**Table 5.** Total calories and selected macro- and micronutrient intake at 2-week and 4 week time points for CRC survivor cohor^*^.

	Control Diet		Navy Bean Powder Diet	
CRC Survivor Study Population	Week 2	Week 4	Week 2	Week 4
Calories (kcal)	1900 ± 373 (1961)	1647 ± 243 (1717)b	1622 ± 287 (1685)	1685 ± 204 (1771)
Protein (g)	76 ± 23 (75)	64 ± 22 (59)b	61 ± 17 (65)	68 ± 17 (62)b
Carbohydrates (g)	226 ± 36 (232)a	200 ± 25 (197)a,b	223 ± 20 (220)a	236 ± 26 (231)a
Fat (g)	77 ± 23 (78)a	65 ± 14 (67)b	55 ± 14 (56)a	58 ± 15 (57)
Fiber (g)	25 ± 7 (23)	20 ± 4 (19)a	30 ± 7 (27)	30 ± 7 (29)a,b

* Values are presented as mean ± SD (median). Medians are included since ranks were compared in the analysis.

a Significance (p≤0.05) between Control and Navy Bean Powder Groups at time point.

b Significance (p≤0.05) at Week 4 compared to Week 2 for specific diet.
